# Supplementary material for: Time‑resolved multi-omic analysis of paclitaxel exposure in human iPSC‑derived sensory neurons unveils mechanisms of chemotherapy‑induced peripheral neuropathy
Source: Cell Death Dis. 2026 Feb 10;17(1):211. doi: 10.1038/s41419-026-08445-2 (PMC12921266; doi:10.1038/s41419-026-08445-2)
Supplement: Supplementary file 15 — Dataset 1. [file 41419_2026_8445_MOESM15_ESM.zip › CDD_Open_Data/05_Protein/02_Intersects_Disco_Plots/Andranik/24h.RNA_vs_protein.pdf]

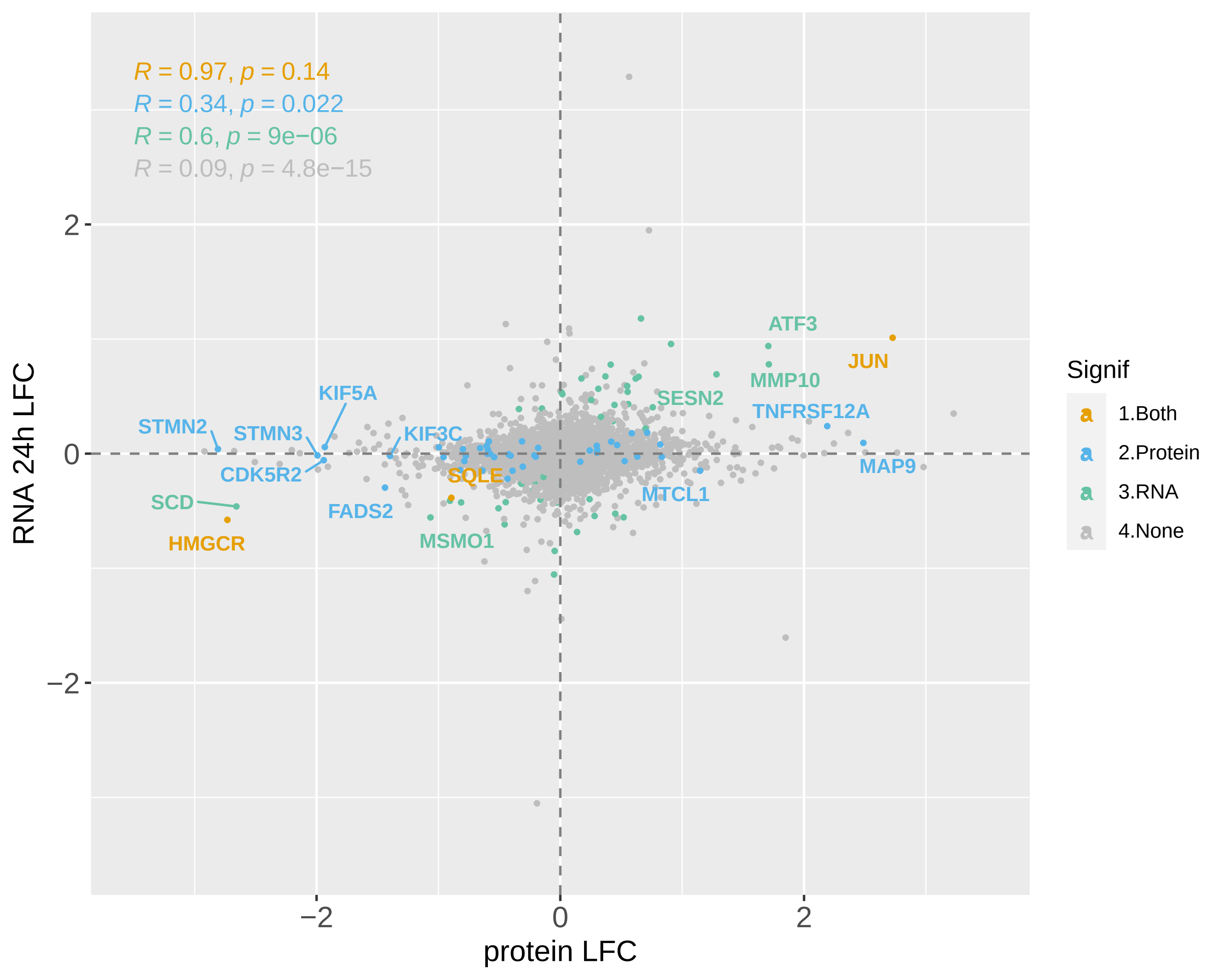

R = 0.97, p = 0.14
R = 0.34, p = 0.022
R = 0.6, p = 9e−06
R = 0.09, p = 4.8e−15
ATF3
JUN
MMP10
SESN2
TNFRSF12A
KIF5A
STMN2
STMN3
KIF3C
CDK5R2
SQLE
MAP9
MTCL1
SCD
FADS2
HMGCR
MSMO1
RNA 24h LFC
protein LFC
2
0
−2
Signif
1.Both
2.Protein
3.RNA
4.None
